# Sequence and Structural Analysis of the Chitinase Insertion Domain Reveals Two Conserved Motifs Involved in Chitin-Binding

**DOI:** 10.1371/journal.pone.0008654

**Published:** 2010-01-13

**Authors:** Hai Li, Lesley H. Greene

**Affiliations:** Department of Chemistry and Biochemistry, Old Dominion University, Norfolk, Virginia, United States of America; Purdue University, United States of America

## Abstract

**Background:**

Chitinases are prevalent in life and are found in species including archaea, bacteria, fungi, plants, and animals. They break down chitin, which is the second most abundant carbohydrate in nature after cellulose. Hence, they are important for maintaining a balance between carbon and nitrogen trapped as insoluble chitin in biomass. Chitinases are classified into two families, 18 and 19 glycoside hydrolases. In addition to a catalytic domain, which is a triosephosphate isomerase barrel, many family 18 chitinases contain another module, i.e., chitinase insertion domain. While numerous studies focus on the biological role of the catalytic domain in chitinase activity, the function of the chitinase insertion domain is not completely understood. Bioinformatics offers an important avenue in which to facilitate understanding the role of residues within the chitinase insertion domain in chitinase function.

**Results:**

Twenty-seven chitinase insertion domain sequences, which include four experimentally determined structures and span five kingdoms, were aligned and analyzed using a modified sequence entropy parameter. Thirty-two positions with conserved residues were identified. The role of these conserved residues was explored by conducting a structural analysis of a number of holo-enzymes. Hydrogen bonding and van der Waals calculations revealed a distinct subset of four conserved residues constituting two sequence motifs that interact with oligosaccharides. The other conserved residues may be key to the structure, folding, and stability of this domain.

**Conclusions:**

Sequence and structural studies of the chitinase insertion domains conducted within the framework of evolution identified four conserved residues which clearly interact with the substrates. Furthermore, evolutionary studies propose a link between the appearance of the chitinase insertion domain and the function of family 18 chitinases in the subfamily A.

## Introduction

### Chitin and Chitinase

Chitin (C_8_H_13_O_5_N)_n_ is a long-chain polymeric polysaccharide of β-glucosamine that forms a hard, semi-transparent material found throughout nature. Chitin is composed of units of N-acetyl-D-glucos-2-amine, which are linked by β-1,4 glycosidic bonds [1]. Hence, it may also be described as cellulose with one hydroxyl group on each monomer replaced by an acetylamine group. Chitin is the main component of the cell walls of fungi [1], the shells and radulae of molluscs, and of the exoskeletons of arthropods, especially crustaceans and insects [2].

The breakdown of chitin is catalyzed by chitinases which hydrolyze it to simple sugars. Chitinases can be divided into two major categories: exochitinases and endochitinases [2], [3]. Exochitinases can be further divided into two subcategories: chitobiosidases, which cleave diacetylchitobiose units from the non-reducing end of the chitin chain, and β-(1,4)-N-acetyl-glucosaminidases (NAGase), which cleave the N-acetylglucosamine (NAG) oligomers, generating NAG monomers. Endochitinases cleave glycosidic linkages randomly at internal sites along the chitin chain, eventually providing a variety of low molecular mass NAG oligomers such as diacetylchitobioses and chitotrioses [2], [3].

Chitinases occur in a wide range of organisms including bacteria, fungi, plants, insects, and animals. Chitinases from bacteria and fungi are extremely important for maintaining a balance between the large amount of carbon and nitrogen trapped in the biomass as insoluble chitin in nature [3], [4]. Chitinases are needed by fungi to disrupt existing cell walls when normal cells divide [5] and chitinases from some plants may be essential in inhibition against fungal pathogens [6]. In insects and crustaceans, chitinases are associated with degradation of old cuticle [7]. Additionally, human chitotriosidase may be important in defence against chitinous pathogens such as *Candida albicans* [8], [9].

Based on amino acid sequence similarity, chitinases are classified into families 18 and 19 of glycoside hydrolases (GH) [10], [11]. The members of the two different families differ in their amino acid sequences, three-dimensional structures, and molecular mechanisms of catalytic reactions [4]. Family 18 chitinases have catalytic domains of triosephosphate isomerase (TIM barrel) fold with a conserved DxDxE motif [12] and catalyze the hydrolytic reaction by substrate-assisted mechanism [13], [14], whereas family 19 chitinases have high percentage of α-helices and adopt the single displacement catalytic mechanism [15], [16]. In family 18 chitinases, the leaving group is protonated by a conserved glutamic acid, the sugar at −1 subsite is distorted into a boat conformation, and an oxazolinium intermediate is stabilized by the sugar N-acetamido group and then hydrolyzed [14], [17]. Family 18 chitinases are widely distributed in five lineages of life; for example, *Thermococcus kodakarensis* [18] in Archaea, *Serratia marcescens* (*S. marcescens*) [19] in Bacteria, *Coccidioides immitis* (*C. immitis*) [20], [21] in Fungi, tobacco [22] in Plantae, and the sandfly [23] and human [24] in Animalia.

### Family 18 Chitinases

**Figure 1:**
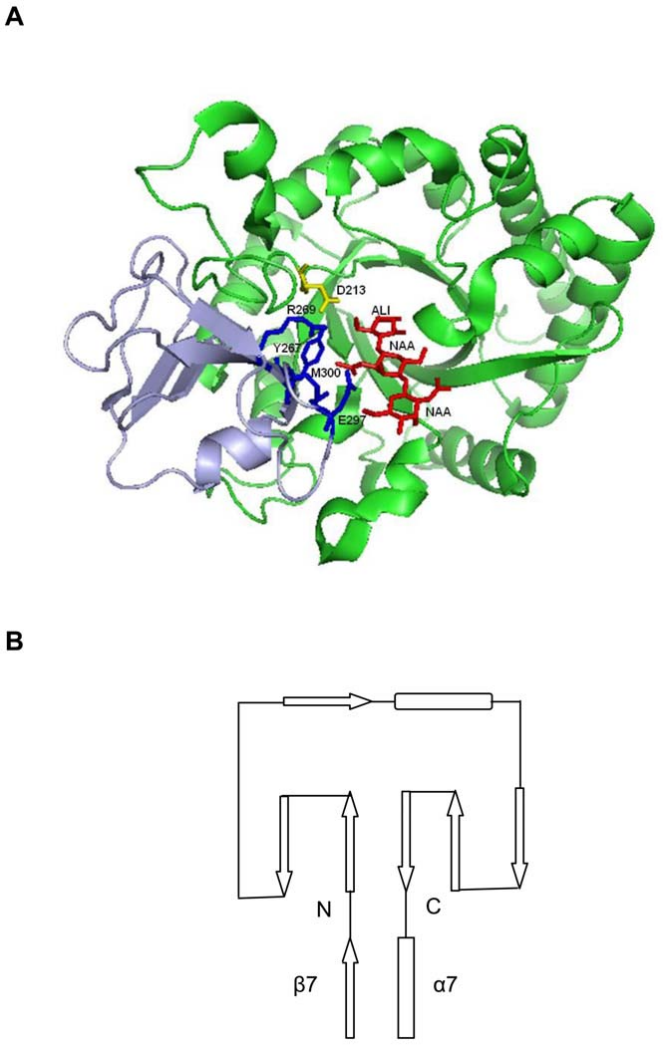
Structural analysis of the CID. (A) Ribbon model of human chitotriosidase (PDB: 1HKM) in complex with the substrate (NAA-NAA-ALI) generated by Pymol, showing the TIM barrel and CID. The helices and strands on the TIM barrel are coloured in green and those on the CID are coloured in light blue. Some residues (Tyr267, Arg269, Glu297, and Met300) in blue on the CID and Asp213 in yellow on the TIM barrel interact with the substrate in red. (B) Schematic representation of the CID between β7 and α7 on the TIM barrel, which is composed of two anti-parallel β-strands followed by one β-strand, one short α-helix, and lastly three anti-parallel β-strands. The arrows indicate β-strands and the rectangles are α-helices. The lines stand for the loops connecting α-helices or β-strands.

Family 18 chitinases can be classified into three subfamilies A, B, and C, in terms of the amino acid sequence similarity [25]. The main structural difference between subfamilies A and B chitinases is that a small α + β domain inserts into the TIM barrel catalytic domain in the subfamily A, while this insertion domain is absent in the subfamily B [26]. For example, human chitotriosidase (PDB code: 1HKM), as a family 18 chitinase in the subfamily A, has a TIM domain and a chitinase insertion domain (CID), which is a module inserted into the TIM barrel (Fig. 1A). In the subfamily A, other additions can occur at N- or C- terminus of the TIM barrel. On the other hand, *S. marcescens* chitinase C (chiC), belonging to the subfamily B, has a catalytic domain, a fibronectin type III-like domain, and a chitin-binding domain [26]. Therefore the presence or absence of the insertion domain appears to be subfamily specific [27]. Examples of family 18 chitinases in the subfamily B are only limited to a few bacteria, such as *S. marcescens* and *Bacillus circulans* (*B. circulans*) [25], [27]. Here we mainly discuss family 18 chitinases in the subfamily A.

The TIM barrel domain consists of an (α/β)_8_-barrel fold and has been found in many different proteins, most of which are enzymes. The TIM barrel domains share low sequence identity and have a diverse range of functions. The specific enzyme activity is determined by the eight loops at the carboxyl end of β-strands [28]. In some TIM barrels, an additional loop from a second domain approaches the active site of the TIM domain and participates in binding and catalysis [28], [29].

The CID is the only family in the CID superfamily and is classified as having an FKBP-like fold in the SCOP database (Fig. 1B) [30]. The CID is composed of five or six anti-parallel β-strands and one α-helix and it inserts between the seventh α-helix and seventh β-strand of the TIM barrel [31]. The CID forms a wall alongside the TIM barrel substrate-binding cleft of chitinase which increases the depth of the cleft. Thus, it is easy to imagine that the substrate-binding cleft of chitinases from the subfamilies B and C is not as deep as that from the subfamily A [27]. Interestingly, some mammalian glycoproteins with various functions also exhibit the fold of a family 18 chitinase, such as human cartilage glycoprotein-39 (HCgp-39), whose structure consists of a TIM domain and a CID [32].

In addition to the TIM domain and the CID, some bacterial chitinases in the subfamily A involved in chitin degradation contain one or two additional domains involved in substrate-binding [33]. For example, *S. marcescens* chitinase A (chiA) (PDB code: 1CTN/1FFR) has an additional N-terminal domain [34] which belongs to the E-set domain superfamily in SCOP, whereas *S. marcescens* chitinase B (chiB) (PDB code: 1E15/1UR9) has one extra C-terminal domain [12] which belongs to the carbohydrate-binding domain superfamily. Removal of such domains often results in enzymes that are still active but show extremely impaired binding to substrates [33], [35]. For example, mutagenesis studies of two tryptophans on the N-terminal domain of chiA resulted in decreased specific hydrolyzing activity thus showing their importance for the hydrolysis of β-chitin [4], [36], [37].

### Four Conserved Residues on the CID May Play an Important Role in Chitinase Function

As known previously, the TIM barrel is considered the catalytic domain in family 18 chitinases [4], [36]. Although a number of previous publications showed interactions between a group of residues on the CID and the enzyme substrate and reported the possible functional significance of the CID [14], [20], [32], [34], [38], the definitive role of the CID in chitinase function has not been completely determined [17], [24], [32]. For example, the functional contribution of the CID is not clear in the case of *S. marcescens* chiA [39]. A previous study showed that by removing the CID from *S. marcescens* chiA, the thermal stability was reduced, the specific activity was decreased, the pH optimum was shifted lower, and the catalytic activity towards long chitin derivatives was lost [39]. However, none of the residues on the CID have been individually mutated. Hence, the role of the specific residues in binding with substrates remains to be identified.

To identify the specific functional residues on the CID, a multiple sequence and structure alignment of this domain was constructed. The sequence search process revealed that this domain exists in a wide range of organisms. Conservation and hydropathy analysis revealed that four conserved residues, constituting two distinct sequence motifs, interact with the substrate. Furthermore, extensive comparisons among different family 18 chitinases demonstrated that the TIM domains + CID can bind long-chain substrates by providing a deep substrate-binding cleft, while this may not be the case for the enzymes with the TIM domain alone. In general additional modules fused to a catalytic domain may play a role in substrate specificity by providing a specific binding site or shaping the active site to recognize a substrate with a different shape or size [40]. We extrapolate that this may be a reason for the insertion of the CID into the TIM barrel. This paper identifies and provides initial computational support for the importance of conserved residues on the CID in chitinase function.

## Results and Discussion

### Structure-Based Sequence Alignment of the CID

**Figure 2:**
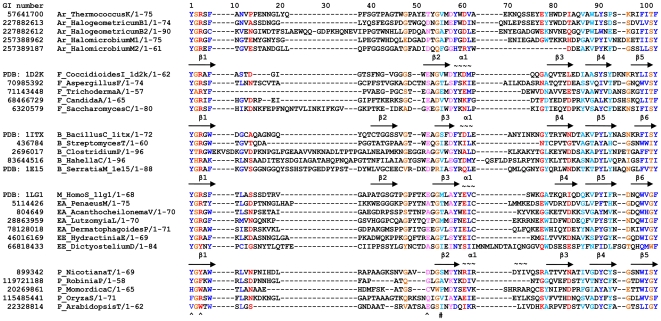
Structure-based multiple sequence alignment of the CID. Hydrophobic positions with high conservation (*C*(i)≥0.45) are coloured in blue and positions with moderate conservation (0.35≤*C*(i)<0.45) are coloured in light blue. Hydrophilic positions with high conservation are coloured in red and positions with moderate conservation are coloured in pink. Neutral positions with high conservation containing mostly glycine, alanine, or proline are coloured in brown, while positions with moderate conservation are not highlighted. ‘∼’ and ‘→’ indicate the sequences in α-helices and β-strands, respectively. The secondary structure of tobacco chitinase CID was predicted by the program of PSIPRED. ‘ ^’ and ‘#’ represent the positions which form hydrogen bonding and the hydrophobic interaction with the substrate, respectively. Smaller alignments can be found in the following references: [24], [31], [33], [78]. The sequences from the following species are listed in the alignment: *T. kodakarensis* KOD1, *Halogeometricum borinquense* DSM 11551, *Halomicrobium mukohataei* DSM 12286, *C. Immitis*, *A. fumigatus*, *Trichoderma atroviride*, *C. albicans* SC5314, *S. cerevisiae*, *B. circulans*, *Streptomyces thermoviolaceus*, *Clostridium paraputrificum*, *Hahella chejuensis* KCTC 2396, *S. marcescens*, *Homo sapiens*, *Penaeus monodon*, *Acanthocheilonema viteae*, *Lutzomyia longipalpis*, *Dermatophagoides pteronyssinus*, *Hydractinia echinata*, *Dictyostelium discoideum* AX4, *Nicotiana tabacum*, *Robinia pseudoacacia*, *Momordica charantia*, *Oryza sativa*, and *Arabidopsis thaliana*. The full genus name and the first letter of species name are shown for each organism in the figure. If two sequences are from one species, a number is added after the species name. All the sequences were obtained from the protein database at the NCBI. Abbreviations: Ar, Archaea; B, Bacteria; F, Fungi; P, Plantae; EE, early eukaryotes; EA, early Animalia; M, mammal.

The representative family 18 chitinases and chitinase-like proteins from plants, bacteria, fungi, and animals whose three-dimensional structures have been determined by X-ray diffraction are listed in Table S1. A multiple sequence alignment of twenty-seven CIDs based on the structures of three model proteins: *B. circulans* chitinase A1 (PDB code: 1ITX), *C. immitis* chitinase (PDB code: 1D2K), and human chitotriosidase (PDB code: 1LG1) was generated by MUSCLE in Jalview (Fig. 2). CIDs from organisms in all five kingdoms are aligned, including Archaea, Bacteria, Fungi, Plantae, and Animalia (Fig. 2). Because of the conservation of the CID, we can identify the sequences boundaries within the multi-domain proteins and further predict the structures of the domain in sequences of family 18 chitinases without solved structures. Further, the secondary structure of the CID of tobacco chitinase is quite similar to those of fungal chitinases, and thus the β-strands and α-helix of plant CIDs can be predicted.

Eight chitinase and chitinase-like structures including the three model chitinases and five more structures (PDB codes: 1LJY, 1FFR, 1UR9, 1KFW, and 1NWT; explained in Table S1) were superimposed on each other based on the CE-MC method (see Fig. S1A). Furthermore, a second and larger sequence alignment with sixty CID sequences was generated using MUSCLE (see Fig. S2).

### Proposed Role of Conserved Residues on the CID

Residues are often conserved in protein families because they either make critical stabilizing interactions or play important functional roles [41]. Additionally, residues important for stability are clustered together in the hydrophobic core and functional residues may be close together in protein-ligand binding sites [41]. Therefore, an analysis of residue conservation is a reasonable approach in which to identify functionally important sites in the CID.

**Figure 3:**
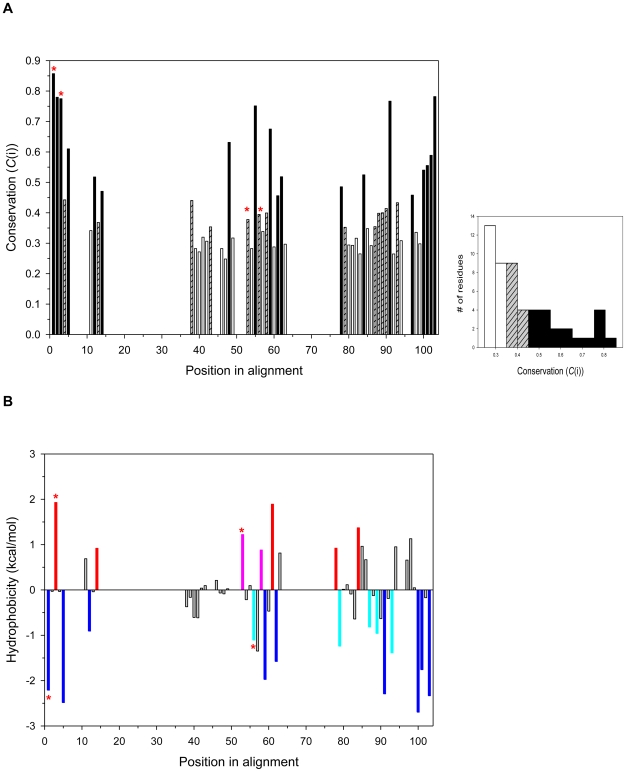
Sequence conservation analysis of the CID. (A) The figure shows the distribution of conservation scores (*C*(i)). Positions with high conservation are represented by black bars (*C*(i)≥0.45), positions with moderate conservation by grey hashed bars (0.35≤*C*(i)<0.45), and positions with less conservation by white bars (*C*(i)<0.35). The conservation values of the positions with more than one gap in the alignment are calculated as zero. Right insert shows the histogram of conservation in terms of the number of positions. Bars annotated with red stars are the conserved residues which may interact with the substrate. (B) The figure shows the average hydropathy profile analysis in the superfamily. Highly conserved hydrophobic positions are represented by blue bars and moderately conserved positions by light blue bars. Highly conserved hydrophilic positions are represented by red bars and moderately conserved positions by pink bars.

Positions of highly and moderately conserved residues (Fig. 3A) and the average hydropathy profile analysis (Fig. 3B) are shown. Our conservation study indicated that there are nine hydrophobic positions with high conservation and five with moderate conservation; five hydrophilic positions with high conservation and two with moderate conservation; and five neutral positions with high conservation and six with moderate conservation (Fig. 2, 3). Among these conserved positions, four on the CIDs in chitinases denoted by PDB codes 1LG1, 1D2K, and 1ITX are proposed to be important for interactions with the substrate, and five for the formation of the hydrophobic core, as well as the stabilization of the domain (Table 1). Interestingly, these four residues fall into two characteristic motifs, one in the N-terminal region and one in the central region, which are termed the YxR motif and the [E/D]xx[V/I] motif, respectively. These two motifs are also conserved in the larger multiple sequence alignment (see Fig. S2) as well as the structural superimpositions (see Fig. S1B). It should be noted that the use of SAM-T08 program also identified the two conserved motifs.

**Table 1:** Conserved residues on the CIDs in chitinases denoted by PDB codes of 1LG1, 1D2K, and 1ITX and their proposed roles.

Code	Interaction with the substrate				Formation of the hydrophobic core				
**1LG1**	Tyr267	Arg269	Glu297	Met300	Tyr303	Val306	Ala312	Val332	Phe334
**1D2K**	Tyr293	Arg295	Glu316	Val319	Tyr322	Met325	Ala330	Ile352	Tyr354
**1ITX**	Tyr338	Arg340	Glu366	Ser369	Phe372	Leu375	Tyr385	Ile407	Tyr409

1LG1: human chitotriosidase; 1D2K: *C. immitis* chitinase; 1ITX: *B. circulans* chitinase A1.

**Figure 4:**
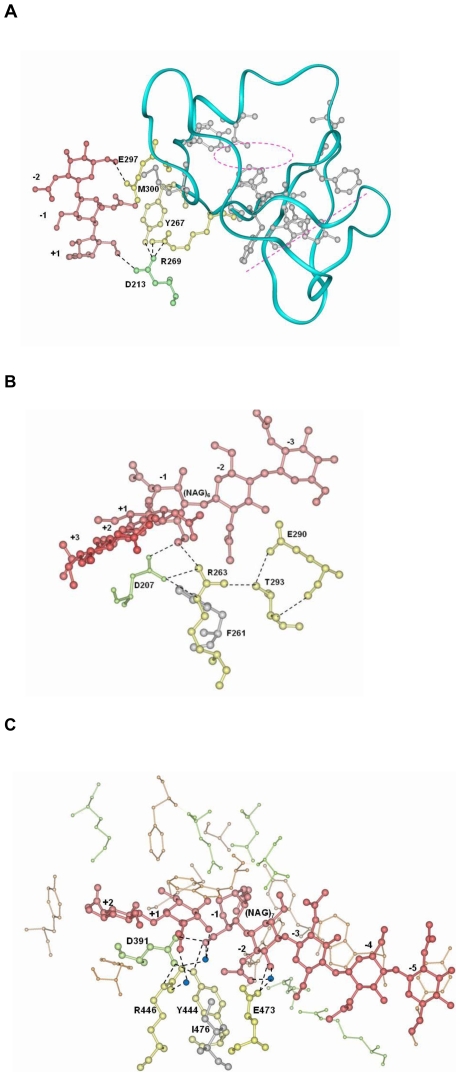
Structures of select family 18 chitinases with their substrates. The TIM barrel residues are coloured in green, the CID residues are in yellow and grey, and substrates are in red. Hydrogen bonds are indicated as dashed lines. (A) The conserved residues on the CID of human chitotriosidase (1HKM) either interact with the substrate, or presumably form a hydrophobic core (Table 1). The α-carbon backbone of the CID is depicted as a blue ribbon. Glu297 on the CID forms a hydrogen bond directly with the substrate while Tyr267 and Arg269 on the CID have hydrogen bonding interactions indirectly through Asp213 on the TIM domain with +1 subsite of the substrate. Tyr267 and Met300 form hydrophobic interactions with the substrate. Some conserved hydrophobic residues in grey appear to form a hydrophobic core which is indicated by a dashed pink circle. Other conserved hydrophobic residues face the straight plane which is indicated by a dashed pink line. They are mostly aromatic and their role is undetermined. (B) Subsites from +3 to −3 in the structure of HCgp-39 (1NWT) are lined up on the main chitin fragment. On the CID of 1NWT, Arg263 forms a hydrogen bond directly with −1 NAG and indirectly via Asp207 on the TIM domain. Phe261 forms a hydrophobic interaction with the oligosaccharide. (C) Residues on *S. marcescens* chiA (1FFR) interact with 7-mer of NAG substrate. Residues in yellow on the CID of 1FFR form hydrogen bonds with the substrate, although some interactions are mediated by Asp391 and water molecules coloured in blue. Ile476 forms a hydrophobic interaction with the substrate. Additional TIM barrel residues involving in hydrogen bonding and hydrophobic interactions are shown in green and brown, respectively. Structures are visualized and analyzed in Insight II. Structural studies analyzing the interactions between the protein and substrate have been previously conducted by other researchers [32], [34], [43].

In the YxR motif, tyrosine and arginine form a pi-cation interaction, which is conserved in all five kingdoms except Plantae. In many family 18 chitinases, a conserved catalytic residue aspartic acid on the TIM barrel (*e.g.* Asp213 in human chitotriosidase, Fig. 4A; Asp391 in *S. marcescens* chiA, Fig. 4C, see [38]), forms an electrostatic interaction with the arginine and hydrogen bonds with both arginine and tyrosine in the motif. The pi-cation interaction, salt bridge, and hydrogen bonding are likely to be important to the structural integrity of the active site including the aspartic acid on the TIM barrel and YxR motif on the CID. These interactions are also conserved in the other family 18 chitinases. *Vibrio harveyi* chitinase A (PDB code: 3B9A) was proposed to catalyze the substrate hydrolysis following the ‘slide and bend mechanism’ as previously described for a long-chain substrate [17]. First, the sugar chain slides forward towards the reducing end distorting the chain especially in −1 NAG, causing it to bend and take up a transient strained boat conformation [14]. Then the twist of the scissile bond, together with the bending of −1 NAG, makes the glycosidic oxygen accessible to the catalytic residue Glu315 for cleavage [17]. This mechanism may also apply to the other family 18 chitinases. In the protein structure 3B9A, Tyr461 and Arg463 in the conserved YxR motif interact with −1 NAG. They also form hydrogen bonds with the conserved catalytic residue Asp392 on the TIM barrel, which interact with three subsites of (NAG)_6_ [17]. *Vibrio harveyi* chitinase A is considered as an endochitinase based on the current literature [42]. However, this is contentious, because its enzyme activity appears to be very similar to that of *S. marcescens* chiA, an exochitinase [42], [43]. In an exochitinase *S. marcescens* chiB, it was proposed that binding of substrate causes the −1 sugar ring to distort to a boat conformation and rotation of Asp142 towards Glu144, thus enabling hydrogen bonding between the acetamido group, Asp142, and Glu144. Later on the oxazolinium ion intermediate was hydrolyzed, leading to protonation of Glu144 and rotation of Asp142, which shares a proton with Asp140 [14]. In another exochitinase *S. marcescens* chiA, after the substrate glycosidic bond is protonated, Asp313 which interacts with Asp311 moves to another position where it interacts with the proton donor residue Glu315, forcing the acetamido group of −1 sugar to rotate. Subsequently, the water molecule that forms hydrogen bonds with Tyr390 and the NH of the acetamido group is displaced to a position which allows hydrolysis to complete [34]. Since the conserved YxR motif on the CID interacts with −1 NAG in *S. marcescens* chiA (see Fig. 4C), it may help cause distortion of the substrate, thus facilitating the cleavage of the glycosidic bonds along the long-chain sugar. Moreover, the YxR motif in chiA forms hydrogen bonds and provides a hydrophilic environment for the catalytic residue Asp391 (see Fig. 4C), which is in a nearly symmetrical position with another catalytic residue Glu315 with respect to the plane of the sugar ring [38]. Interestingly, Asp311, Asp313, and Glu315 in chiA and Asp140, Asp142, and Glu144 in chiB both belong to the conserved TIM barrel DxDxE motif, indicating that their catalytic mechanisms are very similar.

In the substrate-binding site in human chitinase (1HKM), Tyr267 and Arg269 both form hydrogen bonding indirectly by Asp213 with +1 site, and Glu297 directly with −2 site; and Met300 forms a hydrophobic interaction with the substrate (Fig. 4A) [44]. These amino acids, together with neighbouring residues from the TIM domain, may constitute part of the substrate-binding site of the chitinase. Some of the clustered hydrophobic residues (Tyr303, Val306, Ala312, Val332, and Phe334) form a hydrophobic core indicated by the dashed pink circle (Fig. 4A). The roles of the other aromatic residues (Phe271, Tyr324, Phe326, and Trp331) are not exactly known. Interestingly, they face a straight plane indicated by the dashed pink line (Fig. 4A). In human cartilage glycoprotein-39 (HCgp-39) (PDB code: 1NWT), six sugar-binding subsites in the carbohydrate-binding groove across the C-terminal ends of the β-strands of the barrel were identified from −3 to +3 from the non-reducing end (Fig. 4B). The CID also plays a role in sugar-binding because a complex hydrogen bonding network involving conserved residues Arg263, Glu290, and Thr293 on the CID interacts with −1 NAG and Phe261 forms a hydrophobic interaction (Fig. 4B) [32]. Therefore, the other motif [E/D]xx[V/I] also appears to form contacts with substrate.

The other highly conserved neutral positions contain mostly alanine, glycine, or proline; the latter two frequently occur in the structure of β-turns [45] and may be conserved for structural reasons. CID has a large percentage of aromatic residues (*e.g.* 21% in 1ITX). With the exception of some residues which interact with sugar, many of them exist in the hydrophobic core, which may be important for folding and stability. Aromatic residues have been found to play an important role in stabilizing of proteins and peptides [46], [47]. Therefore, the combination of the CID with TIM barrel may increase the thermal stability of the whole enzyme.

### Comparison of GH 18 Proteins with the CID and Those without the CID

Both the NAGase from *Elizabethkingia meningoseptica* (PDB code: 1EOM) and the NAGase from *Streptomyces plicatus* (PDB code: 1EDT) are composed of one TIM domain. They break down the glycosidic bond of (NAG)_2_ to NAG, therefore, they do not have complete chitinolytic activities. In the crystal structure of 1EOM in complex with biantennary octa-saccharide, only the reducing end NAG and two mannoses of the tri-mannose core are in direct contact with the protein [48], while the other sugars extend away from the protein (data not shown). 1EDT hydrolyzes the central β1→4 bond of the diacetylchitobiose core, NAG-(β1-4)-NAG, of asparagine linked oligosaccharides. Unlike the chitinases, the enzyme acts on branched oligosaccharides and has specificities for distinct forms of asparagine-linked oligosaccharides [49], [50].

While only four out of eight units of the substrate interact directly with some residues on 1EOM (Table 2A) [48], in proteins with the TIM domain and CID, a broad network of contacts including hydrophobic interactions and hydrogen bonding exists between the substrate and both the TIM domain and CID. This can be seen, for example, in the analysis of the structure of *S. marcescens* chiA (Fig. 4C, Table 2B) [34].

**Table 2:** Comparison of substrate-binding residues between chitinases with the CID and without the CID.

PDB Code	Subsites for sugar residues	Protein residue			
		Residues on the TIM barrel		Conserved residues on the CID	
		Hydrogen bonding	Hydrophobic interaction	Hydrogen bonding	Hydrophobic interaction
**1EOM (A)**	632	D126, E128, Q211, Y213, Y272	F39		
	633	D18, R20, E245, Y272			
	634	K42, N85, D87, H129	F39		
	641	R20, E245			
	635, 636, 642, 643				
**1FFR (B)**	+2	K369, D391	W275, F396, Y418		
	+1	E315, D391	W275, F316, M388	**R446**	
	−1	D313, E315, D391	Y163, W275, A362, M388, W539	**Y444, R446**	
	−2	E540	W275, W539	**E473**	**I476**
	−3	T276	W167	**E473**	
	−4	R172			
	−5		Y170		

(A) Interactions between select residues on *E. meningoseptica* NAGase (1EOM) and bound polysaccharide. (B) Interactions between some residues on *S. marcescens* chiA (1FFR) and bound substrate (NAG)_7_. The data are adapted from Waddling et al. [48] and Papanikolau et al. [34]. Conserved residues on the CID from our conservation analysis are in bold.

Sun et al. specified that the CID of mouse lectin Ym1 (PDB code: 1E9L) was not involved in the saccharide-binding [51]. Furthermore, they were unable to assign any definitive function for this domain. However, the results of our study indicate that at least four conserved residues on the CID of many chitinases were found to have either hydrogen bonding or hydrophobic interaction with the substrate of more than three units of NAG. While 1E9L was not included in the original structural alignment, a close homologue 1NWT was studied and suggests that the authors may have seen saccharide-binding by the CID if a longer substrate was used.

In CAZy database [52], *S. marcescens* chiA and chiB, *B. circulans* chitinase A1, and *Aspergillus fumigatus* chitinase B (PDB code: 1W9P) are ‘bacterial-type’ exochitinases with a deep or even a tunnel-shaped substrate-binding cleft, formed by the TIM barrel and CID [14], [43], [53]–[54]. *S. marcescens* chiC [43], [54] and ‘plant-type’ chitinases such as hevamine from Para rubber tree (*Hevea brasiliensis*) (PDB code: 1HVQ) [13], *Sc*CTS1 from *Saccharomyces cerevisiae* (PDB code: 2UY2) [55], PPL2 from *Parkia platycephala* seeds (PDB code: 2GSJ) [56], and a hyperthermophilic chitinase from *Pyrococcus furiosus* (PDB code: 2DSK) [57] are endochitinases with a shallow substrate-binding cleft since they lack the CID. Therefore, overall it appears that the CID may enhance the exo-type activity by forming a deep substrate-binding cleft on the top of the TIM barrel [39], [43], [54].

Structures of TIM domain alone, TIM domain + CID, and TIM domain + CID + N- (or C-) terminal domain align very well with their respective counterparts (data not shown). Interactions between residues and substrates are shown in Table 2 for 1EOM (TIM domain alone) and 1FFR (TIM domain + CID). It appears as if more sugar residues interact with amino acid residues when the CID is included in the TIM domain. Therefore, the CID may facilitate stronger association with the substrate, particularly with increasing substrate length. By removing the CID from *S. marcescens* chiA, a processive exochitinase [36], [54], the truncated enzyme appeared to have a shallower tunnel in the catalytic domain than that of the intact enzyme [39] and it resembled the catalytic domain of *S. marcescens* chiC, which acts as a non-processive endochitinase [54]. Therefore, the CID of chiA enhances not only the exo-N,N'-diacetyl-glucosaminidase activity, but also the processivity during the degradation of the polysaccharide chains [39].

### Phylogenetic Analysis of the CID and Evolutionary Scheme of Family 18 Chitinases (Subfamilies A and B)

The ubiquitous TIM barrel fold is adopted by seven enzyme superfamilies, one of which is the TIM barrel GH [40]. The evolutionary relationships between different enzymes with TIM barrel are well studied [40], [58], [59]. Gene duplication, gene fusion, and incremental mutations are three mechanisms by which new functions are created in proteins [40], [60]. Molecular phylogenetic analyses of mammalian GH 18 chitinase and chitinase-like members suggest that active chitinases result from an early gene duplication event, which is followed by mutations, leading to chitinase-like proteins, such as chito-lectins [61]. Comprehensive genomic studies of animal GH 18 proteins showed a similar result [11]. Another phylogenetic analysis of catalytic domain sequences from various organisms showed that sequences of animal, fungi, and bacteria belong to different lineage; however, chitinase genes from lepidopteran insects and baculoviruses originated from bacteria and were maintained through evolution since they transferred laterally [62].

**Figure 5:**
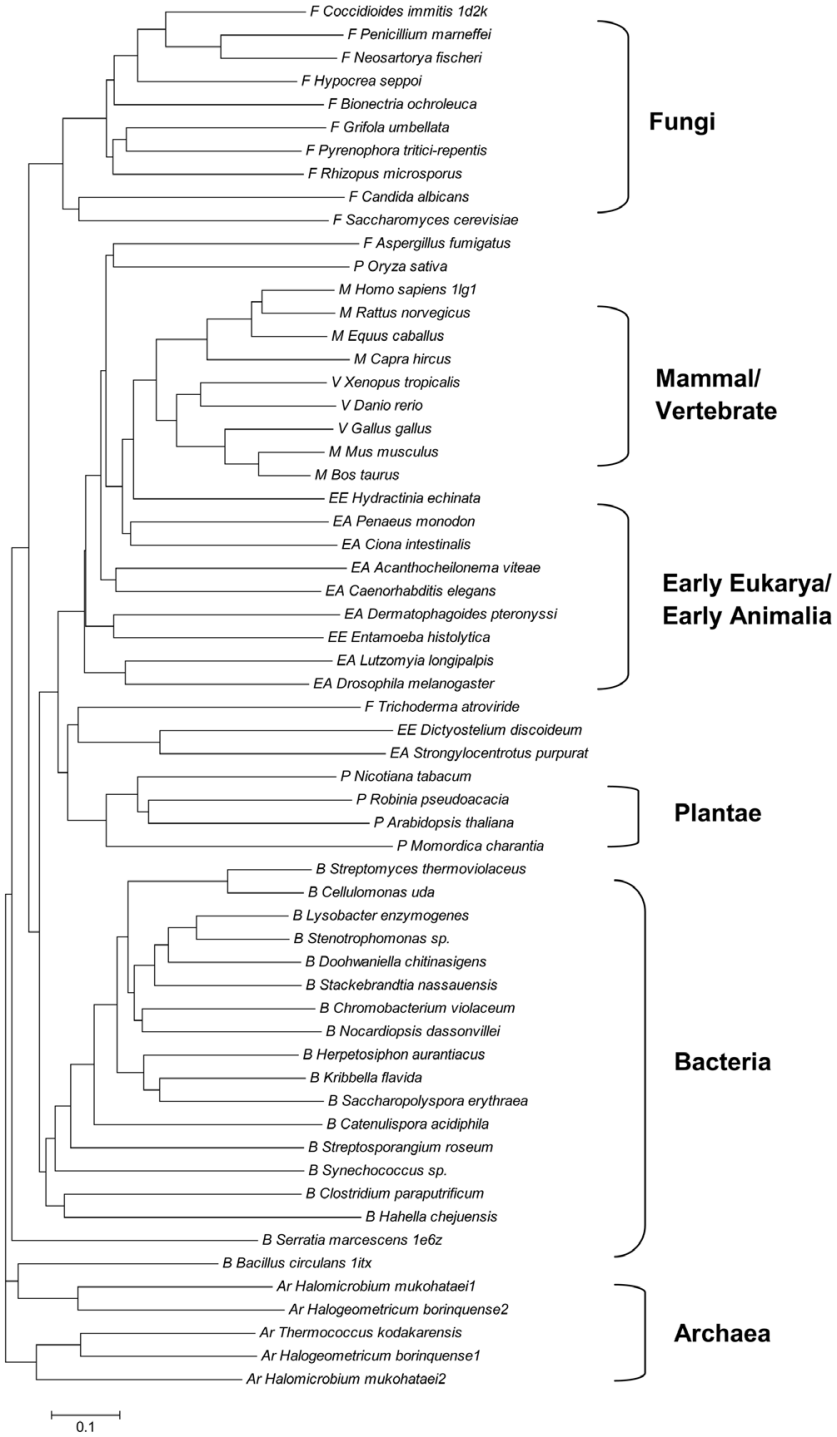
Phylogenetic analysis of the CID sequences from different lineages of organisms. The phylogenetic tree was constructed by the neighbour-joining method based on the CID sequences: five from Archaea, eighteen from Bacteria, twelve from Fungi, five from Plantae, three from early eukaryotes, eight from early Animalia, and nine from vertebrates (V) including six from mammals. The sequence names, corresponding GI numbers, and abbreviations are listed in Fig. 2 and Table S2. All the sequences were obtained from the protein database at the NCBI.

Since the CID sequences are present in all of sixty archaeal, bacterial, and eukaryal genomes in this study, it is possible that the CIDs were present in the Last Universal Common Ancestor (LUCA) [63]. However, no evolutionary study has been conducted on the CID by itself. To establish the phylogenetic relationships between the CIDs from different organisms, a preliminary phylogenetic tree was constructed based on the sixty sequences from five kingdoms (Archaea, Bacteria, Fungi, Plantae, and Animalia) (Fig. 5). Overall, the CID sequences grouped into five major clusters, each representing one kingdom as to be expected. In the cluster of Animalia, members from early eukaryotes and early Animalia branch out earlier than those from vertebrates and mammals.

In the study conducted by Nagano et al. family 18 GH were divided into two functional groups; F4 includes chitinases and F5 includes both hevamine and NAGase [58]. A proposed evolution of the structure and function of family 18 chitinases and chitinase-like proteins in the subfamilies A and B can be potentially described as follows. Due to divergent evolution, a TIM domain line may initially have evolved as hevamine, xylanase inhibitor protein, or seed storage protein (e.g. Concanavalin B) in some higher plants, as well as NAGase in some bacteria. While hevamine has lysozyme/endochitinase function [13], [64], xylanase inhibitor protein [65] and seed storage protein [66] do not have known chitinolytic activity. One possible evolutionary scheme suggests that a TIM barrel evolved to a more potent family 18 chitinase in two routes: *1)* with the incorporation of the CID to form a subfamily A chitinase and *2)* with the other domains (*e.g.* chitin-binding domain) to form a subfamily B chitinase. In the first route, this double-domain chitinase evolved in archaea, bacteria, fungi, plants, and animals, as well as the triple-domain chitinase with the fusion of N- or C- terminal domain in *S. marcescens*. Subsequently, the double-domain chitinase gene was mutated to have novel functions in animals [61].

### Conclusions

Four conserved amino acids identified in this study are proposed to be essential for binding with the substrate and they form two distinguishable sequence motifs. The CID may have inserted into the TIM domain to facilitate orienting and binding to longer (*e.g.*>3) saccharide substrates. Because of the wide distribution in diverse organisms and the high conservation of the CID, we can identify the sequence and predict the structure of this domain in family 18 chitinases in the subfamily A. An evolutionary scheme is presented which places the emergence of the CID in the context of chitinase function; with the addition of the CID leading to an evolutionary shift of the protein from a non-chitinolytic protein, or a NAGase, to a subfamily A or B family 18 chitinase. We also identify a group of conserved hydrophobic residues in the core which we propose are important for folding and structural stability. Research on the role of the CID in function to test this hypothesis can be carried out using a myriad of experimental and computational techniques such as molecular modelling, *in vitro* and *in silico* binding studies coupled to site-directed mutagenesis, enzymatic assays, and crystallization of the holo-proteins.

## Materials and Methods

### Construction of a Multiple Sequence and Structure Alignment of the CID

The CID regions within the structures of three proteins: *B. circulans* chitinase A1 (1ITX), *C. immitis* chitinase (1D2K), and human chitotriosidase (1LG1) were used as query sequences in PSI-BLAST to search for distant relatives. They represent chitinases within the kingdoms of Bacteria, Fungi, and Animalia, respectively. A plant or archaeal structure was not available at the time however the PSI-BLAST searches did identify plant and archaeal chitinases for inclusion in our study. An initial multiple sequence alignment was made using MUSCLE - multiple protein sequence alignment program in Jalview (Java alignment editor) [67], [68]. In the searched sequences, some from close relatives have high identities >40% (data not shown). Five sequence relatives from each of the five kingdoms and two from early eukaryotes with sequence identities less than 40% were chosen to make the final twenty-seven representatives of the CID superfamily. The alignment was created in order to enhance sequence variability and in this way, only the key conserved residues for structure, folding, and function could be identified. The boundary of the CID in each sequence was identified by aligning with the three model chitinases and the domain was further extracted from each chitinase sequence.

An initial structure alignment containing the CIDs from 1ITX, 1D2K, and 1LG1 was generated with the online CE-MC - multiple protein structure alignment program [69]. The initial sequence alignment was compared with the initial structure alignment, and adjusted in Jalview to ensure the sequences with unknown structures were properly aligned with the known structures. Since no structure from plant is available, the secondary structure of tobacco chitinase CID was predicted by the program of PSIPRED [70], and the other sequences were aligned with it thereafter.

To verify our sequence and structure alignment, eight representatives of family 18 chitinases structures (1HKM, 1LJY, 1ITX, 1D2K, 1FFR, 1UR9, 1KFW, and 1NWT) were superimposed with CE-MC method [69]. In addition to the twenty-seven CID sequences from Archaea, Bacteria, Fungi, Plantae, and Animalia, thirty-three more sequences from Bacteria, Fungi, and Animalia (see Table S2) were acquired from searches of the protein database using the PSI-BLAST program. A larger multiple sequence alignment of sixty sequences was generated using MUSCLE in Jalview, without being edited according to the three model structures. Furthermore, the SAM-T08 program was employed to search for the conserved residues in the CID (http://compbio.soe.ucsc.edu/SAM_T08/T08-query.html) [71].

### Conservation and Hydropathy Analysis

The number of each residue in each position was calculated and analyzed by SigmaPlot 10.0 (SYSTAT Software Inc.). The entropy value was calculated by the following equation:
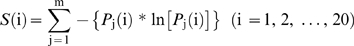



*P*_j_(i) is the fractional occurence of amino acid type j at each site, and m is the number of amino acid types used in the particular analysis [72]. Furthermore, conservation was calculated by the following equation: *C*(i) = 1−*S*(i)/ln(m) [73]. The positions with conservation values greater than 0.45 were considered to be highly conserved; the positions with conservation values between 0.35 and 0.45 were considered to be moderately conserved; and those positions with conservation values lower than 0.35 were considered to be less conserved [73]. The positions which have more than one gap are considered non-conserved and therefore have a value of zero. Hydropathy was calculated by the following equation: hydropathy = sum of the number of each amino acid * hydrophobicity of that amino acid. The hydrophobicity scale of Nozaki and Tanford was used for our studies [74].

Select structures from the designated family 18 chitinases in SCOP (http://scop.mrc-lmb.cam.ac.uk/scop/) and CAZy (http://www.cazy.org/fam/GH18.html) were chosen to compare the structure and function of chitinases and chitinase-like proteins (see Table S1). Protein data bank (PDB) files were obtained from SCOP and RCSB (http://www.rcsb.org). All PDB files were visualized and analyzed in either Insight II, version 2005 (Accelrys, CA), Pymol, version 0.99 (DeLano Scientific, CA), or Rasmol, version 2.7. Hydrogen bond calculations and van der Waals radii were determined with Insight II.

### Phylogenetic Analysis of the CID

In order to investigate the evolutionary relationship of the CID sequences from different lineages of life, the ClustalW2 program (http://www.ebi.ac.uk/Tools/clustalw2/index.html) was performed with the sixty CID sequences, because the program can produce a multiple sequence alignment of divergent sequences and Cladogram or Phylogram to visualize the evolutionary relationships [75]. The phylogenetic tree was constructed using the neighbour-joining algorithm as described by Saitou and Nei [76]. The tree was visualized and drawn with MEGA version 4.0.2 software [77].

## Supporting Information

Figure S1 Superimposition of eight family 18 chitinases and chitinase-like structures (1HKM, 1LJY, 1ITX, 1D2K, 1FFR, 1UR9, 1KFW, and 1NWT) and the two conserved motifs on the CIDs. The structures were superimposed with the CE-MC method. (A) The eight structures 1HKM, 1LJY, 1ITX, 1D2K, 1FFR, 1UR9, 1KFW, and 1NWT are coloured in red, orange, yellow, green, blue, cyan, purple, and black, respectively. The aligned, blue, and cyan parts are TIM domain + CID, N-terminal domain on 1FFR, and C-terminal domain on 1UR9, respectively. (B) The two residues in the YxR motif are shown in red and orange, respectively; and the two residues in the [E/D]xx[V/I] motif are shown in yellow and blue, respectively.
(4.45 MB TIF)

Figure S2 The larger multiple sequence alignment of sixty CID sequences from various species. The alignment was generated by MUSCLE in Jalview. It is not edited according to the three model structures. The two conserved motifs YxR and [E/D]xx[V/I] are highlighted in the red frames and the four conserved positions are labelled with red stars. The species names and GI numbers refer to Table S2.
(3.57 MB TIF)

Table S1 List of twenty-one family 18 chitinases and chitinase-like proteins from plants, bacteria, fungi, and animals. Structures in bold are described and compared in the text.
(0.06 MB DOC)

Table S2 List of the sequence names, species name, and GI numbers of thirty-three more CID sequences that are included in the phylogenetic tree (Fig. 5) and the larger multiple sequence alignment (Fig. S2).
(0.05 MB DOC)
