# Supplementary figures and images for: Sequence and Structural Analysis of the Chitinase Insertion Domain Reveals Two Conserved Motifs Involved in Chitin-Binding
Source: PLoS One. 2010 Jan 13;5(1):e8654. doi: 10.1371/journal.pone.0008654 (PMC2805709; doi:10.1371/journal.pone.0008654)

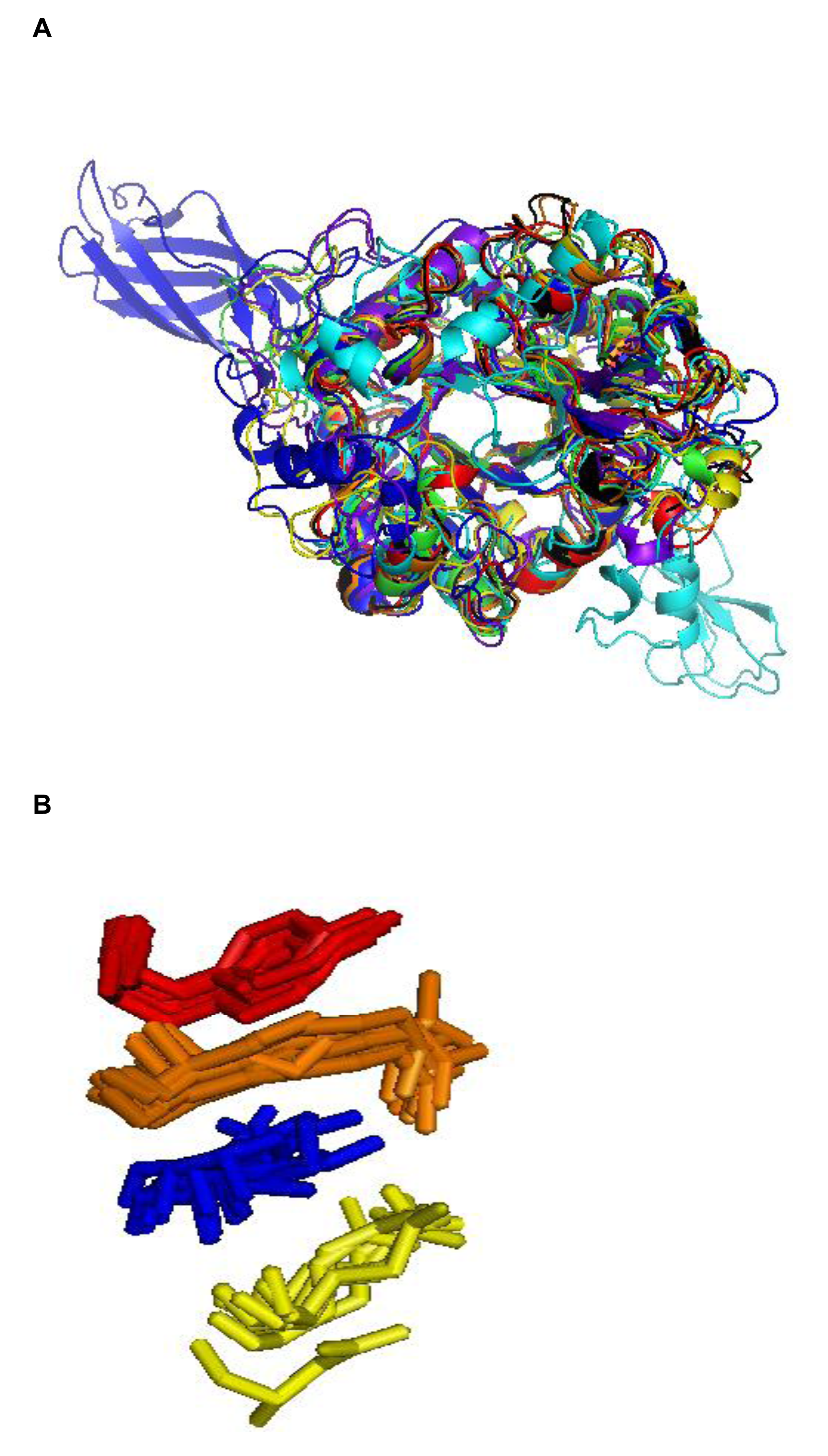

Supplement: Figure S1 — Superimposition of eight family 18 chitinases and chitinase-like structures (1HKM, 1LJY, 1ITX, 1D2K, 1FFR, 1UR9, 1KFW, and 1NWT) and the two conserved motifs on the CIDs. The structures were superimposed with the CE-MC method. (A) The eight structures 1HKM, 1LJY, 1ITX, 1D2K, 1FFR, 1UR9, 1KFW, and 1NWT are coloured in red, orange, yellow, green, blue, cyan, purple, and black, respectively. The aligned, blue, and cyan parts are TIM domain + CID, N-terminal domain on 1FFR, and C-terminal domain on 1UR9, respectively. (B) The two residues in the YxR motif are shown in red and orange, respectively; and the two residues in the [E/D]xx[V/I] motif are shown in yellow and blue, respectively. (4.45 MB TIF) [file pone.0008654.s001.tif]

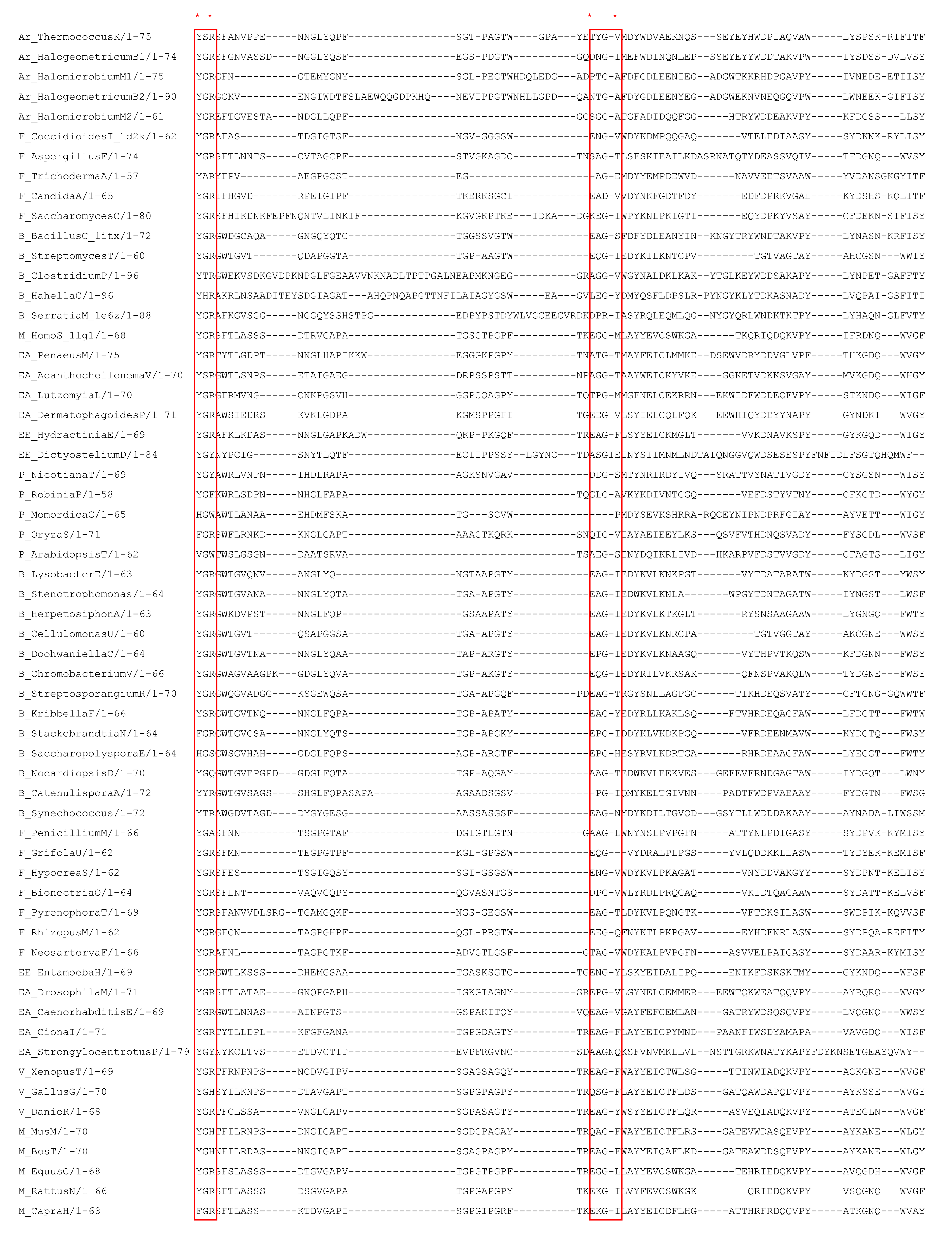

Supplement: Figure S2 — The larger multiple sequence alignment of sixty CID sequences from various species. The alignment was generated by MUSCLE in Jalview. It is not edited according to the three model structures. The two conserved motifs YxR and [E/D]xx[V/I] are highlighted in the red frames and the four conserved positions are labelled with red stars. The species names and GI numbers refer to Table S2. (3.57 MB TIF) [file pone.0008654.s002.tif]
