# Supplementary material for: Sequence and Structural Analysis of the Chitinase Insertion Domain Reveals Two Conserved Motifs Involved in Chitin-Binding
Source: PLoS One. 2010 Jan 13;5(1):e8654. doi: 10.1371/journal.pone.0008654 (PMC2805709; doi:10.1371/journal.pone.0008654)
Supplement: Table S1 — List of twenty-one family 18 chitinases and chitinase-like proteins from plants, bacteria, fungi, and animals. Structures in bold are described and compared in the text. (0.06 MB DOC) [file pone.0008654.s003.doc]

**Table S1.**

| **PDB Code** | **Species** | **Name/**  **Kingdom** | **With CID?** | **Other domain** | **Ligand** | **Function** |
| --- | --- | --- | --- | --- | --- | --- |
| 1HVQ | [*H. brasiliensis*](http://www.rcsb.org/pdb/search/smartSubquery.do?smartSearchSubtype=TreeQuery&t=11&n=51536) | Para rubber tree | No |  | 3 NAG | [Hevamine A (endochitinase/lysozyme](http://www.rcsb.org/pdb/search/smartSubquery.do?smartSearchSubtype=TreeQuery&t=11&n=51535)) |
| 1TA3 | [*Triticum aestivum*](http://www.rcsb.org/pdb/search/smartSubquery.do?smartSearchSubtype=TreeQuery&t=11&n=89479) | Wheat | No |  | NAG, EDO (scatter) | [Xylanase inhibitor protein](http://www.rcsb.org/pdb/search/smartSubquery.do?smartSearchSubtype=TreeQuery&t=11&n=89478) |
| 1CNV | [*Canavalia ensiformis*](http://scop.mrc-lmb.cam.ac.uk/scop/data/scop.b.d.b.j.f.e.html) | Jack bean | No |  |  | Concanavalin B, seed storage protein |
| 1NAR | *Vicia narbonensis* | Purple broad vetch | No |  |  | Narbonin, seed storage protein |
| **1EOM** | [*E. meningoseptica*](http://www.rcsb.org/pdb/search/smartSubquery.do?smartSearchSubtype=TreeQuery&t=11&n=51542) | [Bacteri](http://www.rcsb.org/pdb/search/smartSubquery.do?smartSearchSubtype=TreeQuery&t=11&n=51542)um | No |  | 3 NAG, 3 MAN, 2 GAL | [NAGase](http://www.rcsb.org/pdb/search/smartSubquery.do?smartSearchSubtype=TreeQuery&t=11&n=51540) |
| 1EDT | [*S. plicatus*](http://www.rcsb.org/pdb/search/smartSubquery.do?smartSearchSubtype=TreeQuery&t=11&n=51543) | [Bacteri](http://www.rcsb.org/pdb/search/smartSubquery.do?smartSearchSubtype=TreeQuery&t=11&n=51542)um | No |  |  | [NAGase](http://www.rcsb.org/pdb/search/smartSubquery.do?smartSearchSubtype=TreeQuery&t=11&n=51540) |
| 2EBN | [*E. meningoseptica*](http://www.rcsb.org/pdb/search/smartSubquery.do?smartSearchSubtype=TreeQuery&t=11&n=51542) | [Bacteri](http://www.rcsb.org/pdb/search/smartSubquery.do?smartSearchSubtype=TreeQuery&t=11&n=51542)um | No |  |  | NAGase |
| **1ITX** | [*B. circulans*](http://www.rcsb.org/pdb/search/smartSubquery.do?smartSearchSubtype=TreeQuery&t=11&n=75070) | [Bacteri](http://www.rcsb.org/pdb/search/smartSubquery.do?smartSearchSubtype=TreeQuery&t=11&n=51542)um | Yes |  |  | Chitinase A1 |
| **1FFR/1CTN** | [*S. marcescens*](http://www.rcsb.org/pdb/search/smartSubquery.do?smartSearchSubtype=TreeQuery&t=11&n=49234) | [Bacteri](http://www.rcsb.org/pdb/search/smartSubquery.do?smartSearchSubtype=TreeQuery&t=11&n=51542)um | Yes | [N-terminal](http://www.rcsb.org/pdb/search/smartSubquery.do?smartSearchSubtype=TreeQuery&t=11&n=49233) | 7 NAG | Chitinase A |
| **1UR9/1E15** | [*S. marcescens*](http://www.rcsb.org/pdb/search/smartSubquery.do?smartSearchSubtype=TreeQuery&t=11&n=49234) | [Bacteri](http://www.rcsb.org/pdb/search/smartSubquery.do?smartSearchSubtype=TreeQuery&t=11&n=51542)um | Yes | C-terminal | NAG, GDL, PHJ | Chitinase B |
| 1KFW | [*Arthrobacter* sp*.*](http://scop.mrc-lmb.cam.ac.uk/scop/data/scop.b.d.b.j.f.ba.html) | [Bacteri](http://www.rcsb.org/pdb/search/smartSubquery.do?smartSearchSubtype=TreeQuery&t=11&n=51542)um | Yes |  |  | Psychrophilic chitinase B |
| **3B9A** | *V. harveyi* | Bacterium | Yes | [N-terminal](http://www.rcsb.org/pdb/search/smartSubquery.do?smartSearchSubtype=TreeQuery&t=11&n=49233) | 6 NAG | Chitinase A |
| **1D2K** | [*C. immitis*](http://www.rcsb.org/pdb/search/smartSubquery.do?smartSearchSubtype=TreeQuery&t=11&n=51549) | Fungus | Yes |  |  | Chitinase |
| 1WNO/1W9P | *A. fumigatus* | Fungus | Yes |  | NAG, NDG | Chitinase B |
| **1LG1/1HKM** | *H. sapiens* | Human | Yes |  | 2 NAA, 1 ALI | Chitotriosidase |
| 3FXY | *H. sapiens* | Human | Yes |  |  | Acidic Mammalian Chitinase |
| 1E9L | *Mus musculus* | Mouse | Yes |  | NAG | Mammalian lectin |
| **1NWT** | *H. sapiens* | Human | Yes |  | 8 NAG | Cartilage gp39 |
| 1JND | [*Drosophila melanogaster*](http://scop.mrc-lmb.cam.ac.uk/scop/data/scop.b.d.b.j.f.cc.html) | Fruit fly | Yes |  | NAG, MAN | Disc growth factor-2 |
| 2DPE | [*Ovis aries*](http://scop.mrc-lmb.cam.ac.uk/scop/data/scop.b.d.b.j.f.bh.html) | Sheep | Yes |  | 2 NAG, 3 MAN | Signal processing protein |
| 1LJY | *Capra hircus* | Goat | Yes |  | NAG | Mammary gland protein |
