# Supplementary material for: Sequence and Structural Analysis of the Chitinase Insertion Domain Reveals Two Conserved Motifs Involved in Chitin-Binding
Source: PLoS One. 2010 Jan 13;5(1):e8654. doi: 10.1371/journal.pone.0008654 (PMC2805709; doi:10.1371/journal.pone.0008654)
Supplement: Table S2 — List of the sequence names, species name, and GI numbers of thirty-three more CID sequences that are included in the phylogenetic tree (Fig. 5) and the larger multiple sequence alignment (Fig. S2). (0.05 MB DOC) [file pone.0008654.s004.doc]

**Table S2.**

| **Sequence name** | **Species name** | **GI number** |
| --- | --- | --- |
| B_LysobacterE | *Lysobacter enzymogenes* | 50429005 |
| B_Stenotrophomonas | *Stenotrophomonas sp.* SKA14 | 254524159 |
| B_HerpetosiphonA | *Herpetosiphon aurantiacus* ATCC 23779 | 159899269 |
| B_CellulomonasU | *Cellulomonas uda* | 17865808 |
| B_DoohwaniellaC | *Doohwaniella chitinasigens* | 6649589 |
| B_ChromobacteriumV | *Chromobacterium violaceum* ATCC 12472 | 34499695 |
| B_StreptosporangiumR | *Streptosporangium roseum* DSM 43021 | 229855168 |
| B_KribbellaF | *Kribbella flavida* DSM 17836 | 227377795 |
| B_StackebrandtiaN | *Stackebrandtia nassauensis* DSM 44728 | 229863927 |
| B_SaccharopolysporaE | *Saccharopolyspora erythraea* NRRL 2338 | 134102989 |
| B_NocardiopsisD | *Nocardiopsis dassonvillei* subsp. dassonvillei DSM 4311 | 229205033 |
| B_CatenulisporaA | *Catenulispora acidiphila DSM 44928* | 256395265 |
| B_Synechococcus | *Synechococcus sp.* RCC307 | 148242001 |
| F_PenicilliumM | *Penicillium marneffei* ATCC 18224 | 212534216 |
| F_GrifolaU | *Grifola umbellata* | 28436151 |
| F_HypocreaS | *Hypocrea seppoi* | 220701877 |
| F_BionectriaO | *Bionectria ochroleuca* | 88696577 |
| F_PyrenophoraT | *Pyrenophora tritici-repentis* Pt-1C-BFP | 189188560 |
| F_RhizopusM | *Rhizopus microsporus* var. oligosporus | 1565203 |
| F_NeosartoryaF | *Neosartorya fischeri* NRRL 181 | 119470878 |
| EE_EntamoebaH | *Entamoeba histolytica* HM-1:IMSS | 67472835 |
| EA_CaenorhabditisE | *Caenorhabditis elegans* | 17551250 |
| EA_DrosophilaM | *Drosophila melanogaster* | 45550474 |
| EA_CionaI | *Ciona intestinalis* | 167830427 |
| EA_StrongylocentrotusP | *Strongylocentrotus purpuratus* | 115608306 |
| V_XenopusT | *Xenopus (Silurana) tropicalis* | 4262194 |
| V_GallusG | *Gallus gallus* | 45383307 |
| V_DanioR | *Danio rerio* | 41055329 |
| M_MusM | *Mus musculus* | 12597291 |
| M_BosT | *Bos taurus* | 27807261 |
| M_EquusC | *Equus caballus* | 219689080 |
| M_RattusN | *Rattus norvegicus* | 119120779 |
| M_CapraH | *Capra* *hircus* | 66361429 |
